# Predicting factors for chronic colonization of *Pseudomonas aeruginosa* in bronchiectasis

**DOI:** 10.1007/s10096-019-03675-z

**Published:** 2019-08-31

**Authors:** A. Pieters, M. Bakker, R. A. S. Hoek, J. Altenburg, M. van Westreenen, J. G. J. V. Aerts, M. M. van der Eerden

**Affiliations:** Erasmus Medical Center, Rotterdam, The Netherlands

**Keywords:** *Pseudomonas aeruginosa*, Bronchiectasis, Mortality, Chronic colonization

## Abstract

About 25% of the patients with bronchiectasis are likely to develop a chronic colonization with *Pseudomonas aeruginosa*. A better understanding of predictors of acquiring *Pseudomonas* within the patient population may facilitate future focused research. The aim of this retrospective observational study was to investigate predicting factors for *P. aeruginosa* colonization in patients with bronchiectasis. This was a single-center retrospective cohort study using a bronchiectasis database which consisted of 211 patients with bronchiectasis. Data were collected for demographic details, etiology, spirometry, microbiology data, maintenance medication use, exacerbation frequency, hospital admission rate, and FACED and Bronchiectasis Severity Index (BSI) score. Two hundred eleven patients were identified from our bronchiectasis database. Overall, 25% of the patients (*n* = 53) had a chronic colonization with *P. aeruginosa*. Seventeen patients (8%) died in a 5-year follow-up period of whom 7 (41%) had a chronic *P. aeruginosa* colonization (*p* > 0.05). After multiple regression analysis, *P. aeruginosa*-positive patients were significantly associated with an older age (> 55 years) (*p* = 0.004), the use of hypertonic saline (0.042), and inhalation antibiotics (< 0.001). Furthermore, the presence of PCD (*p* < 0.001) and post-infectious etiology (*p* < 0.001) as underlying causes were significantly associated with *P. aeruginosa* colonization. We observed that independent predictors for *P. aeruginosa* colonization were age > 55 years, hypertonic saline, and PCD, and post-infectious etiology as underlying causes of bronchiectasis. Since prevention of *P. aeruginosa* colonization is an important aim in the treatment of bronchiectasis, more attention could be directed to these groups at risk for *Pseudomonas* colonization.

## Introduction

About 25% (range from 9 to 33%) of the patients with bronchiectasis are likely to develop a chronic colonization with *Pseudomonas aeruginosa* [1–3]. The available evidence to date suggests that *P. aeruginosa* colonization in bronchiectasis is associated with poorer outcome in terms of hospital admissions, exacerbation frequency, and mortality [3–7]. However, the independent patient characteristics for *P. aeruginosa* colonization are not well known. A better understanding of the predictors of acquiring *Pseudomonas* within the patient population may facilitate future focused research and identify patient subgroups which may benefit from intensive management to prevent chronic colonization as *P. aeruginosa*.

The aim of this retrospective observational study was to investigate predicting factors for *P. aeruginosa* colonization in patients with bronchiectasis.

## Methods

### Design

This was a single-center retrospective cohort study using a bronchiectasis database of the department of pulmonary medicine of the Erasmus University Medical Center, Rotterdam, The Netherlands. Patients who visited the outpatient clinic between January 2012 and December 2016 were included. The study cohort consisted of 211 patients with bronchiectasis. Eligible patients were at least 18 years old and had been diagnosed with bronchiectasis according to the typical findings of abnormal dilatation of the bronchi found on high resolution CT scan together with a clinical syndrome of cough, sputum production, and airway infection. Patients with a confirmed diagnosis of cystic fibrosis by genotyping and/or sweat testing were excluded. The study has been approved by the medical ethical committee of the Erasmus MC Rotterdam.

### Procedures

The majority of patients were seen every 3 months at the outpatient clinic. During a visit, a standard spirometry (FVC, FEV1, and FEV1/FVC) was performed and sputum samples were collected. The database consisting of individual case notes was reviewed for the collection of demographic details, details on the diagnosis of bronchiectasis, assessment of comorbidities (chronic obstructive pulmonary disease (COPD), asthma, cardiac comorbidities, neurologic disorders, diabetic mellitus, and chronic renal impairment), smoking history, lung function test results in the baseline and follow-up, microbiology data, maintenance medication use, exacerbation frequency, hospital admission rate, and FACED and Bronchiectasis Severity Index (BSI) scores [4, 5]. Underlying etiology had been identified according to the methods described by Pasteur et al. [1, 8].

Patient data sets were derived from the hospital visit database until data capture point (December 2016) or death.

### Sputum cultures

Sputum samples were regularly obtained during outpatient clinic visits or during hospital admissions and processed according to the American Society of Microbiology guidelines [9]. We regarded a sputum sample as representative when more than 25 polymorphonuclear leucocytes and less than 10 squamous cells per low-power field were identified by Gram stain. We established breakpoints according to the European Committee on Antimicrobial Susceptibility Testing [10].

### Spirometry

Spirometry was performed according to ERS/ATS standards in all patients using a standard spirometer at routine clinical visits. The best FEV1% predicted value recorded in each year was used in the analysis.

### Outcome

For the determination of the impact of chronic *P. aeruginosa* colonization, patients with at least two *P. aeruginosa*-positive cultures, 3 months apart over the course of 12 months, were compared with patients without this definition of chronic *P. aeruginosa* colonization. Group comparisons were performed with the collected data as have been described in the Procedures section.

### Statistical analysis

Statistical analysis was performed using the Statistical Package for the Social Sciences (SPSS), version 21.0 (SPSS Inc., Chicago, IL, USA). Continuous variables were presented as a mean standard deviation (SD) for normal distribution or median with interquartile range (25th–75th percentile) for non-normal distribution.

The incidence of exacerbation and hospital admission is summarized as a per-person per-year rate. Differences in exacerbations between groups were analyzed with the use of nominal parametric data using Student’s *t* test and a non-parametric Kruskal-Wallis test. The chi-squared test (Fisher’s exact test, when appropriate) was used to compare proportions. All *p* values were nominal and two-sides and were not adjusted for multiple comparisons. *p* values less than 0.05 were considered significant.

Chronic *P. aeruginosa* colonization was analyzed as an indicator variable (a patient did have or did not have a chronic *P. aeruginosa* colonization) to determine factors associated with chronic *P. aeruginosa* colonization, fitting univariate models with the use of logistic regression. We used multinomial logistic regression to more fully characterize the associations between selected baseline factors and chronic *P. aeruginosa* colonization. All variables that were explored in the univariate logistic regression were considered in the multivariate model (all different variables). A conservative significance threshold of 0.04 was used to determine the qualification of data for entry into or deletion from the model. The odds ratio (OR), 95% confidence interval, and *p* value were computed for each of presenting predictors.

## Results

### Patient population

Two hundred eleven patients were identified from our bronchiectasis database between January 2012 and December 2016. The time-dependent variables were evaluated in 2016; they were comparable with the other observational study years (Table 1). The cohort analysis showed that 54% of the patients were male (*n* = 113) and that the median age was 60 years. In the majority of cases, the cause of bronchiectasis was attributed to idiopathic and post-infectious diseases, in 31% and 20% respectively. One hundred thirty patients (63%) had never smoked. Of the current or previous smokers, the pack-years interquartile range varied between 7 and 31 with a median of 18.5. HRCT scan showed that the majority of cases (*n* = 94; [45%]) had three or more lobes affected. The distribution of the severity score according to the FACED scale was 48% (*n* = 99) mild, 32% (*n* = 67) moderate, and 20% (*n* = 41) severe, and according to the BSI was 40% (*n* = 82) mild, 24% (*n* = 50) was moderate, and 36% (*n* = 75) was severe.

**Table 1:** Patient characteristics

Parameters	Overall cohort (*n* = 211)	Chronic *P. aeruginosa* colonization (*n* = 53)	No chronic *P. aeruginosa* colonization (*n* = 158)	*p* value#
Age (years)	60 (45–74)	67 (57–77)	58 (43–73)	0.237
Age > 55 years	158 (75%)	40 (74%)	92 (58%)	*0.025*
Male: female	113 (54%)	30 (57%)	83 (53%)	0.607
BMI m kg^−2^	23 (20–26)	24 (20–28)	23 (20–26)	0.765
FEV1 predicted at baseline	79 (63–96)	75 (58–93)	81 (65–97)	0.184
Comorbid condition				
Cardiologic comorbidities	50 (24%)	11 (21%)	39 (25%)	0.546
Neurologic disorders	24 (11%)	6 (11%)	18 (12%)	0.977
Diabetic mellitus	19 (9%)	3 (6%)	16 (10%)	0.414*
Chronic renal impairment	22 (11%)	3 (6%)	19 (12%)	0.185
Smoker				*0.032*
Never	130 (63%)	39 (75%)	91 (58%)	
Ever or current	78 (37%)	13 (25%)	65 (42%)	
Smoking pack-years	18.5 (6.5–30.5)	26 (13–39)	15 (3–28)	
Exacerbation frequency				< *0.001*
None exacerbations	58 (32%)	6 (12%)	52 (40%)	
One exacerbation	51 (29%)	12 (26%)	39 (30%)	
≥ Two exacerbations	70 (39%)	29 (62%)	41 (31%)	
Hospital admissions				< *0.001*
None hospital admissions	140 (78%)	28 (60%)	112 (85%)	
≥ One hospital admissions(s)	39 (22%)	19 (40%)	20 (15%)	
Cause				
Idiopathic	65 (31%)	12 (22%)	54 (34%)	0.117
Post-infectious	43 (20%)	18 (34%)	25 (16%)	*0.005*
Primary or secondary immunodeficiency	39 (19%)	5 (9%)	34 (22%)	0.050
PCD	17 (8%)	10 (19%)	7 (4%)	0.002*
COPD	14 (7%)	2 (4%)	11 (7%)	0.524*
ABPA	10 (5%)	3 (6%)	7 (4%)	0.714*
Severe asthma	10 (5%)	–	10 (6%)	0.069*
Aspiration/reflux	8 (4%)	2 (4%)	6 (4%)	–
IBD	3 (1%)	–	3 (2%)	–
Yellow nail syndrome	1 (1%)	1 (2%)	–	–
Job syndrome	1 (1%)	–	1 (1%)	–
Radiology (HRCT scan)				
One lobe affected	43 (20%)	7 (13%)	36 (23%)	0.134
Two lobes affected	74 (35%)	13(25%)	61 (39%)	0.063
≥ Three lobes affected	94 (45%)	33 (62%)	61 (39%)	*0.003*
Pathogens (other than *P. aeruginosa*)				
*Staphylococcus aureus*	26 (22%)	6 (15%)	20 (25%)	0.210
*Haemophilus influenzae*	11 (9%)	5 (13%)	6 (8%)	0.503*
*Aspergillus fumigatus*	23 (19%)	10 (25%)	13 (16%)	0.251
*NTM*	6 (5%)	3 (4%)	3 (8%)	0.399*
*Stenotrophomonas maltophilia*	9 (8%)	4 (10%)	5 (6%)	0.479*
Medications				
Hypertonic saline - inhalation	85 (48%)	30 (64%)	55 (42%)	*0.009*
Inhalation antibiotics	31 (15%)	20 (42%)	11 (8%)	< *0.001*
Inhaled corticosteroids	19 (11%)	8 (17%)	11 (8%)	0.105*
Inhaled corticosteroids/long-acting Beta-agonists	97 (54%)	68 (50%)	29 (62%)	0.229
Macrolides maintenance	136 (65%)	44 (83%)	92 (58%)	*0.001*
FACED scale				< *0.0001*
Mild	99 (48%)	8 (15%)	91 (59%)	
Moderate	67 (32%)	23 (43%)	44 (29%)	
Severe	41 (20%)	22 (42%)	19 (12%)	
BSI scale				**<** *0.0001*
Mild	82 (40%)	3 (6%)	79 (51%)	
Moderate	50 (24%)	9 (17%)	41 (27%)	
Severe	75 (36%)	41 (77%)	34 (22%)	

Data are presented as number of patients, unless otherwise stated

Data are displayed as median with 25th and 75th percentile in parentheses

*COPD*, chronic obstructive pulmonary disease; *ABPA*, allergic bronchopulmonary aspergillosis; *PCD*, primary ciliary dyskinesia; *IBD*, inflammatory bowel disease; *NTM*, non-tuberculous mycobacteria

# Pa colonization versus others, analyzed with Mann-Whitney *U* test for continuous variables and a chi-square test for categorical variables

*Fisher’s test

The median (± interquartile range) follow-up period from 2012 was 47 months (± 13); 17 patients (8%) died in a 5-year follow-up period of whom 7 (41%) had a chronic *P. aeruginosa* colonization (*p* > 0.05).

Overall, 25% of the patients (*n* = 53) had a chronic colonization with *P. aeruginosa.*

With a univariate analysis, we analyzed difference in proportions between *P. aeruginosa*-positive and *P. aeruginosa*-negative patients, using all available baseline variables in the whole cohort, showed in Table 1. Patients with *P. aeruginosa* colonization had significantly more lobes affected on HRCT scan compared with *P. aeruginosa*-negative patients (*p* = 0.003). The etiology seems to be different, with significantly more frequent primary ciliary dyskinesia (PCD) (*p* = 0.002) and post-infectious etiology (*p* = 0.005) as underlying causes of bronchiectasis compared with patients without *P. aeruginosa* colonization. Furthermore, an older age (> 55 years) was associated with *P. aeruginosa* colonization (*p* = 0.025). Other significant differences between *P. aeruginosa* colonization and patients without *P. aeruginosa* colonization were a higher rate of smoking status (*p* = 0.032), a more frequent use of maintenance therapy with macrolides (*p* = 0.001), and a worse outcome in the clinical scoring systems according to the FACED scale (*p* < 0.0001) and BSI scale (*p* < 0.0001). Subsequently, patients with *P. aeruginosa* colonization had a significantly increased exacerbation frequency (*p* < 0.001) and an increased number of hospital admissions (*p* < 0.001).

In multiple regression analysis, variables that were positive after univariate analysis were used as predictors for *P. aeruginosa* colonization. In the *Pseudomonas* colonization group, exacerbation frequency and hospital admissions, shown to be positive after the univariate analysis, were not taken into account because we considered it the consequence and not the cause of *Pseudomonas* colonization. Factors that were independently associated with the presence of *P. aeruginosa* colonization, on the basis of the multiple regression model, are shown in Table 2. *P. aeruginosa*-positive patients were significantly associated with an older age (> 55 years) (*p* = 0.004), the use of hypertonic saline (0.042), and inhalation antibiotics (< 0.001). Furthermore, the presence of PCD (*p* < 0.001) and post-infectious etiology (*p* < 0.001) as underlying causes was significantly associated with *P. aeruginosa* colonization.

**Table 2:** Factors associated with *Pseudomonas aeruginosa* colonization (in the multivariate model)

Parameters	Multivariable analysis	
	OR (95% C.I.)	*p* value
Age > 55 years	0.150 (0.042–0.540)	*0.004*
HRCT (> 3 lobes affected)	0.516 (0.188–1.418)	0.199
PCD	0.040 (0.007–0.288)	< *0.001*
Post-infectious cause	0.048 (0.013–0.186)	< *0.001*
Macrolide maintenance	0.950 (0.321–2.901)	0.950
Hypertonic saline inhalation	0.309 (0.100–0.959)	*0.042*
Inhalation antibiotics	0.059 (0.016–0.220)	< *0.001*

## Discussion

In our observational study of patients with bronchiectasis, we identified patient characteristics which were associated with the presence of persistent *P. aeruginosa* colonization. Patient characteristics predicting for *P. aeruginosa* colonization were older age, use of inhalation antibiotics and hypertonic saline, and PCD and post-infectious etiology as underlying causes of bronchiectasis.

*Pseudomonas aeruginosa* is an important pathogen in bronchiectasis and functions as a marker for disease severity. In our study, 25% of the patients had a chronic colonization with *P. aeruginosa.* The average prevalence of *P. aeruginosa* colonization in a recently published European survey was 15% [7]. In other studies, prevalence ranged from 9 to 33% [1, 3]. In a subpopulation of our study, a remarkable high prevalence of *P. aeruginosa* infection was present in patients with PCD (58%) and in post-infectious (42%) causes of bronchiectasis. A comparable high rate of *P. aeruginosa* colonization in PCD was reported in a prevalence study performed by Shah et al. [11]. They found a prevalence of *P. aeruginosa* colonization in 44% of PCD patients compared with 22% in an adult bronchiectasis cohort. A recent study in patients with PCD showed a prevalence of *Pseudomonas* colonization in 27.6% of the patient population [12]. Other predicting factors for *Pseudomonas* colonization were the use of inhalation antibiotics, which was not a surprise since inhalation antibiotics were solely given to patients with *Pseudomonas* colonization. The use of hypertonic saline also had been associated with *Pseudomonas* colonization, which has not been described before. It is hard to determine whether the use of hypertonic saline is a cause or consequence of *Pseudomonas* colonization. In other studies, a significant association between worse radiological imaging and *P. aeruginosa* colonization has been reported [3–5, 13, 14]. However, we could not show this observation in our study. Another study also described a low FEV1% predicted value and the presence of polymicrobial colonization as independent predictors of *P. aeruginosa* colonization [6].

In the study performed by Araujo et al., the presence of *P. aeruginosa* colonization in patients with bronchiectasis was associated with a worse quality of life and with an increase in exacerbation frequency and hospital admissions [7]. They also showed that patients with frequent exacerbations, who were colonized with *P. aeruginosa*, had an increased risk of mortality. Finch et al. also showed in a review that *P. aeruginosa* colonization was associated with a significant increase in mortality risk [3]. However, we observed that there was no relation between *P. aeruginosa* colonization and increased mortality risk. This might be attributed to the smaller sample size of our study. In total, 17 patients (8%) with bronchiectasis died in a 5-year observational period, of which 7 were colonized with *P. aeruginosa*. This percentage of mortality is much lower than the 20.5% in a 5-year follow-up as described by Goeminne et al. [15]. This could be explained by the presence of a low percentage of COPD patients (7%) in our population, since Goeminne et al. showed that patients with COPD patients with bronchiectasis had a significant higher mortality rate [15]. Furthermore, Araujo et al. showed that one of the risk factors for mortality was the number of exacerbations [7]. In our study, there were a high percentage of patients receiving long-term macrolide treatment (65%) to prevent exacerbations compared with the Goeminne study with a macrolide treatment percentage of 40% [15]. Other recent studies do not describe the percentage of macrolide treatment, which in our opinion is important to mention concerning the impact on frequency of exacerbations and prognosis [6, 7, 15].

This study included several important limitations. First, because it is a retrospective study, there is a risk of bias. Second, since this was a single-center study, we included a relatively small number of patients. Third, we defined *Pseudomonas* colonization when at least two *P. aeruginosa*-positive cultures were present which were 3 months apart over the course of 12 months. There is a lack of international criteria for defining *Pseudomonas* colonization in bronchiectasis. In cystic fibrosis (CF), the European consensus definition for chronic *Pseudomonas* infection is the presence of at least three positive cultures over ≥ 6 months with at least a 1-month interval between the samples [16]. The Leeds criteria for chronic *Pseudomonas* infection in CF refer to patients in whom sputum cultures were positive for *Pseudomonas* in > 50% of the samples during 12 months [17]. Our experience is that it is more difficult to culture *Pseudomonas* in bronchiectasis patients than in patients with cystic fibrosis. Therefore, we used a more practical definition; since in clinical practice, sputum cultures were not always available.

To conclude, we observed that independent predictors for *P. aeruginosa* colonization were age > 55 years, the use of hypertonic saline, and PCD and post-infectious etiology as underlying causes of bronchiectasis. Since prevention of *P. aeruginosa* colonization is an important aim in the treatment of bronchiectasis, more attention could be directed to these groups at risk for *Pseudomonas* colonization.

## References

[1] Pasteur MC, Helliwell SM, Houghton SJ, Webb SC, Foweraker JE, Coulden RA (2000). An investigation into causative factors in patients with bronchiectasis. Am J Respir Crit Care Med.

[2] Ho PL, Chan KN, Ip MS, Lam WK, Ho CS, Yuen KY (1998). The effect of Pseudomonas aeruginosa infection on clinical parameters in steady-state bronchiectasis. Chest..

[3] Finch S, McDonnell MJ, Abo-Leyah H, Aliberti S, Chalmers JD (2015) A comprehensive analysis of the impact of Pseudomonas aeruginosa colonization on prognosis in adult bronchiectasis. Ann Am Thorac Soc 12(11):1602–1611 10.1513/AnnalsATS.201506-333OC 26356317

[4] Martinez-Garcia MA, de Gracia J, Vendrell Relat M, Giron RM, Maiz Carro L, de la Rosa Carrillo D (2014). Multidimensional approach to non-cystic fibrosis bronchiectasis: the FACED score. Eur Respir J.

[5] Chalmers JD, Goeminne P, Aliberti S, McDonnell MJ, Lonni S, Davidson J (2014). The bronchiectasis severity index. An international derivation and validation study. Am J Respir Crit Care Med.

[6] McDonnell MJ, Jary HR, Perry A, MacFarlane JG, Hester KL, Small T (2015). Non cystic fibrosis bronchiectasis: a longitudinal retrospective observational cohort study of Pseudomonas persistence and resistance. Respir Med.

[7] Araújo David, Shteinberg Michal, Aliberti Stefano, Goeminne Pieter C., Hill Adam T., Fardon Thomas C., Obradovic Dusanka, Stone Glenda, Trautmann Marion, Davis Angela, Dimakou Katerina, Polverino Eva, De Soyza Anthony, McDonnell Melissa J., Chalmers James D. (2018). The independent contribution of Pseudomonas aeruginosa infection to long-term clinical outcomes in bronchiectasis. European Respiratory Journal.

[8] Pasteur MC, Bilton D, Hill AT (2010). British Thoracic Society Non CFBGG. British Thoracic Society guideline for non-CF bronchiectasis. Thorax.

[9] HD I Clinical microbiology procedures handbook, 3th edn. American Society of Microbiology Press, Washington DC

[10] EUCAST. European Committee on Antimicrobial Susceptibility Testing (EUCAST). (Clinical breakpoints. http://www.eucast.org/clinical_breakpoints/)

[11] Shah A, Shoemark A, MacNeill SJ, Bhaludin B, Rogers A, Bilton D (2016). A longitudinal study characterising a large adult primary ciliary dyskinesia population. Eur Respir J.

[12] Cohen-Cymberknoh M, Weigert N, Gileles-Hillel A, Breuer O, Simanovsky N, Boon M (2017). Clinical impact of Pseudomonas aeruginosa colonization in patients with Primary Ciliary Dyskinesia. Respir Med.

[13] Loebinger MR, Wells AU, Hansell DM, Chinyanganya N, Devaraj A, Meister M (2009). Mortality in bronchiectasis: a long-term study assessing the factors influencing survival. Eur Respir J.

[14] Miszkiel KA, Wells AU, Rubens MB, Cole PJ, Hansell DM (1997). Effects of airway infection by Pseudomonas aeruginosa: a computed tomographic study. Thorax.

[15] Goeminne PC, Nawrot TS, Ruttens D, Seys S, Dupont LJ (2014). Mortality in non-cystic fibrosis bronchiectasis: a prospective cohort analysis. Respir Med.

[16] Doring G, Conway SP, Heijerman HG (2000). Antibiotic therapy against *Pseudomonas aeruginosa* in cystic fibrosis: a European consensus. Eur Respir J.

[17] Lee TWR, Brownlee KG, Conway SP, Denton M, Littlewood JM (2003). Evaluation of a new definition for chronic*Pseudomonas aeruginosa* infection in cystic fibrosis patients. J Cystic Fibros.

